# GateMeClass: Gate Mining and Classification of cytometry data

**DOI:** 10.1093/bioinformatics/btae322

**Published:** 2024-05-22

**Authors:** Simone Caligola, Luca Giacobazzi, Stefania Canè, Antonio Vella, Annalisa Adamo, Stefano Ugel, Rosalba Giugno, Vincenzo Bronte

**Affiliations:** Veneto Institute of Oncology IOV-IRCCS, Padova, Italy; Section of Immunology, Department of Medicine, University of Verona, Verona, Italy; Veneto Institute of Oncology IOV-IRCCS, Padova, Italy; Section of Immunology, Azienda Ospedaliera Universitaria Integrata (AOUI), Verona, Italy; Section of Immunology, Department of Medicine, University of Verona, Verona, Italy; Section of Immunology, Department of Medicine, University of Verona, Verona, Italy; Department of Computer Science, University of Verona, Verona, Italy; Veneto Institute of Oncology IOV-IRCCS, Padova, Italy

## Abstract

**Motivation:**

Cytometry comprises powerful techniques for analyzing the cell heterogeneity of a biological sample by examining the expression of protein markers. These technologies impact especially the field of oncoimmunology, where cell identification is essential to analyze the tumor microenvironment. Several classification tools have been developed for the annotation of cytometry datasets, which include supervised tools that require a training set as a reference (i.e. reference-based) and semisupervised tools based on the manual definition of a marker table. The latter is closer to the traditional annotation of cytometry data based on manual gating. However, they require the manual definition of a marker table that cannot be extracted automatically in a reference-based fashion. Therefore, we are lacking methods that allow both classification approaches while maintaining the high biological interpretability given by the marker table.

**Results:**

We present a new tool called GateMeClass (Gate Mining and Classification) which overcomes the limitation of the current methods of classification of cytometry data allowing both semisupervised and supervised annotation based on a marker table that can be defined manually or extracted from an external annotated dataset. We measured the accuracy of GateMeClass for annotating three well-established benchmark mass cytometry datasets and one flow cytometry dataset. The performance of GateMeClass is comparable to reference-based methods and marker table-based techniques, offering greater flexibility and rapid execution times.

**Availability and implementation:**

GateMeClass is implemented in R language and is publicly available at https://github.com/simo1c/GateMeClass

## 1 Introduction

Future advances in biology and medicine strongly depend on the ability to analyze high-dimensional single-cell datasets. In this context, cytometry is an important tool for studying cell composition and dynamics by examining the expression of protein markers. The traditional approach adopted for analyzing cytometry data is the manual gating, a sequential selection of cellular populations of interest based on the intensity level of specific markers. In flow cytometry, a combination of fluorescent molecules conjugated to antibodies that specifically recognize either extracellular or intracellular markers is used to identify different cell populations in a unique cell suspension. In mass cytometry (CyTOF) (Bandura *et al.* 2009), the cells are labeled using metal-tagged antibodies, nebulized, and vaporized to form ion clouds. After a purification step, the remaining ions are quantified using a time-of-flight (TOF) mass spectrometer to determine the intensity level of each marker. Manual gating is sequentially applied to determine the expression pattern of specific cell populations looking at scatter plots. Manual gating has some limitations: (i) large experiments, where several parameters are evaluated, might be time-consuming; (ii) multi-dimensional data are analyzed in 2D plots by considering two parameters at a time; (iii) manual analysis is susceptible to operator-dependent variability.

Unsupervised cluster-based approaches have emerged to increase automation. Cluster analysis can be faster and less biased under certain conditions or with appropriate algorithmic choices compared to manual gating. Each cluster is usually annotated by examining the expression of cell markers after projecting the data into a low-dimensional space using algorithms such as t-SNE and UMAP. Clustering is also used to identify new cell subsets. Considering its importance, several clustering methods have been proposed for the analysis of cytometry data (Qiu *et al.* 2011, Becher *et al.* 2014, Shekhar *et al.* 2014, Levine *et al.* 2015, Van Gassen *et al.* 2015). However, clustering has some limitations. First, it usually depends on user-defined parameters (e.g. number of clusters) that determine the result. Second, the annotation of clusters for assigning cell labels is time-consuming.

Supervised or semisupervised approaches have been also proposed to improve the degree of automation in cell annotation. This category includes machine learning methods that annotate cytometry data starting from a reference set of annotated cell types such those based on deep learning such as DGCyTOF (Li *et al.* 2017, Cheng *et al.* 2022) and linear discriminant analysis (LDA) (Abdelaal *et al.* 2019). The advantages of DGCyTOF include its high accuracy, the handling of batch effects taking advantage of a calibration step and the integration of a visualization toolkit. LDA is technically simpler and more computationally efficient, however, it does not handle possible batch effects. A disadvantage of deep learning methods is their black-box nature that could make biological interpretation and further validation with traditional manual gating difficult. A common disadvantage of DGCyTOF and LDA is that they require a training set. In fact, while the availability of reference cytometry data is continuously growing, the training data must be representative of the current experiment in terms of cell populations and must have the same panel of protein markers used in the experiment. This is not always possible because the markers selected for a cytometry experiment are limited and highly context-specific, therefore potentially not comparable between different datasets.

One category of tools that does not require training data for cell annotation are those which classify the cells based on a user-defined marker table, such as ACDC (Lee *et al.* 2017) and MP (Ji *et al.* 2018). These approaches input a table containing a list of cell types with a “pseudo gating strategy” in which a marker is positive (+) if the cell type expresses that marker and negative (−) otherwise. This information is used for classification. The main advantages of these methods include their high biological interpretability due to the use of a marker table that mimics the gating strategies used in manual gating and their independence from a reference dataset. One limitation of these methods is that the construction of a marker table requires extensive knowledge of cell markers and is time-consuming. Furthermore, these tools do not manage medium expression, i.e. different levels of expression of a given marker in terms of fluorescence intensity or mass signals. The expression of a marker might show little variations among cell populations. Such little variations might be crucial to distinguish cell populations with different biological properties. Since the current methods based on a marker table use a binary definition of marker expression, they cannot detect cell populations with intermediate intensity of a marker. Finally, these methods have an average higher computational time compared to machine learning methods.

Here, we present a method called GateMeClass (Gate Mining and Classification) that overcomes some of the limitations of existing methods by allowing both supervised and semisupervised classification of cytometry data and managing medium expression of cell markers. GateMeClass performs the cytometry data classification using a marker table that can be specified manually or can be learned from an annotated dataset. We compared GateMeClass with some of the methods of supervised classification (i.e. LDA, DGCyTOF) and semisupervised classification (i.e. MP, ACDC) for cytometry data using different cytometry datasets to demonstrate its applicability to real biological data. GateMeClass obtained comparable performance in terms of accuracy and F1-score compared to the other classifiers.

## 2 Materials and methods

**Figure 1.**
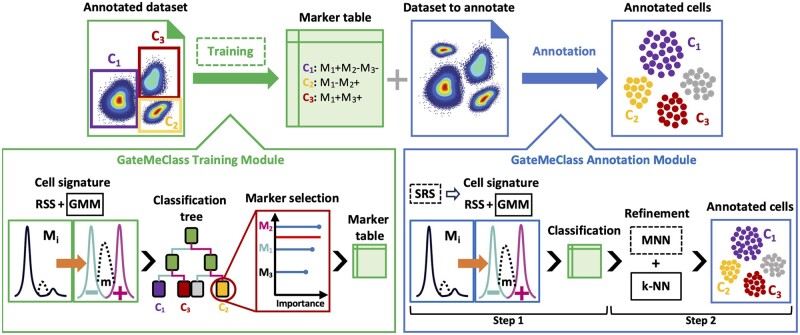
GateMeClass workflow. In the training module (left panel), a marker table is extracted from an annotated dataset using Gaussian mixture models (GMM) and feature selection. In the annotation module (right panel), after an optional simple random sampling (SRS) step, GMM modeling, MNNs, and k-NN are used to annotate the cells based on a marker table. Dashed lines indicate optional features of the tool

GateMeClass is composed by two main modules: the training module, and the annotation module (Fig. 1). Both modules share a computational procedure determining the cell signature, i.e. if a cell has positive/hi (+), medium/mid (m), negative/lo (−), or any (*) expression for each protein marker. GateMeClass assumes that the training and annotation modules are executed individually in a homogeneous dataset. In the training module (Fig. 1, left panel), GateMeClass extracts a marker table from an annotated reference dataset using a feature selection step that exploits a classification tree. In the annotation module (Fig. 1, right panel), GateMeClass inputs a marker table that can be defined manually or obtained through the training module. The marker table is used to annotate the current cytometry dataset comparing the cell signatures with a dictionary of possible signatures generated by GateMeClass parsing the marker table. Finally, the cell annotations can be refined through a procedure based on k-NN and mutual nearest neighbors (MNN) algorithms (Fig. 1, right panel).

Sections 2.1, 2.2, and 2.3 describe, respectively, the computational procedures for generating the cell signature, the training module, and the annotation module of GateMeClass. Section 2.4 reports the evaluation metrics used to measure the performance.

### 2.1 Generating a cell signature

Both training and annotation modules are based on a core procedure that determines whether a cell has a high, medium, or low expression for each marker (Fig. 1, bottom left panel and bottom right panel). The core statistical model used to determine the range of expression of each marker is the Gaussian mixture model (GMM). In GateMeClass, we use the univariate formulation of GMM to determine whether a cell has a high, medium, or low expression of a marker. Briefly, a univariate GMM with G components (or clusters) is defined as follows:

(1) $$p\left(x\right)=\sum_{i=i}^{G}\phi_{i}N\left(x|\mu_{i},\sigma_{i}\right)$$

where N denotes a normal distribution, $\mu_{i}$ and $\sigma_{i}$ denote the center and standard deviation of the i*-*th Gaussian component. $\phi_{i}$ is the weight of the i*-*th component with the constraint that $\sum_{i=1}^{G}\phi =1$ to normalize the total probability to 1. The parameters $\mu_{i}$, $\sigma_{i}$, and $\phi_{i}$ are commonly estimated using an expectation maximization (EM) algorithm.

The GMM model is used to assign all the cells to the corresponding cluster (high, medium, and low marker) based on the maximum posteriori component assignment probability. Due to its flexibility, GMM and other mixture models have been successfully used in the past to identify cell populations in cytometry data (Chan *et al.* 2008, Lo *et al.* 2008, Finak *et al.* 2009, Pyne *et al.* 2009, Naim *et al.* 2010). To attenuate the effect of skewness of cytometry data on GMM clustering, we implemented a method based on ranked set sampling (RSS) (Wolfe 2012) as a preprocessing step in the GMM modeling. RSS is a sampling technique introduced by McIntyre (1952) as a method to ensure truly representative data collection compared to simple random sampling (SRS). In RSS, at first, $m^{2}\;$random points are generated from the population. Second, the $m^{2}$ units are arranged into m sets of size m following an appropriate ranking. Third, for each ranked set, one unit is selected as follows: for the first set, the first ranked unit is selected, for the second set, the second unit is selected, and so on. Finally, m units among the $m^{2}$ points are selected. This procedure is iterated n times where n is the number of cycles*.* RSS observations are the set of points in the same ranking position of different ranked sets and represent different order statistics (or strata) of the population. In GateMeClass, the RSS observations are used differently. To obtain a better resolution of the tails of the distribution of the marker, the RSS procedure is parameterized with m=2 to divide the distribution of the marker into two strata: “leftmost,” i.e. lowest order statistics, and “rightmost,” i.e. greatest order statistics. Next, if the distribution of the marker is positively skewed, the rightmost strata of the distribution will be selected for GMM; otherwise, the leftmost will be selected. The skewness of the distribution of a marker is calculated using the R package “moments” (https://CRAN.R-project.org/package=moments). This procedure counteracts the asymmetry of the distribution of a marker by performing a “zoom” of the elected tail. For each marker, a sampling of the total cells is generated through the above procedure and used for generating the cell signature through GMM. GateMeClass is based on the GMM implementation of the “mclust” package (Scrucca *et al.* 2016), which offers two types of parameterizations of the GMM model: variable (V) and equal (E) variance. The choice of the variance parameter strictly depends on the data to model. For each marker, GateMeClass determines the number of clusters (i.e. number of Gaussians) G to use based on the marker table’s entries. If a marker is defined only as positive or negative, a GMM model with G=2 will be fitted; while if a medium expression is also defined, a GMM with G=3 will be fitted. The marks “−,” “m,” or “+” will be attributed considering the order of the centers of the Gaussian components. When “m” is present the mark “+” represents the higher expression. At the end of this process, GateMeClass will produce a complete matrix of cell signatures.

Algorithm 1. Training module of GateMeClass.

1: GateMeClass_training(reference_dataset):

2: for each cell c_i_ :

3: S_i_ ← Cell_Signature(c_i_ )

4: endfor

5: MT ← Create_Empty_Marker_Table()

6: for each pair of cell types (c_i_ , c_j_ ):

7: T ← Build_Classification_tree(S)

8: VI ← Variable_Importance(T)

9: M_k_ ← Select_Top_Important_Marker(VI)

10: M_e_ ← Set_Marker_Expression(M_k_ )

11: if M_e_ is not present in entry MT_i_ or MT_j_ :

12: Update_Marker_Table(M_e_ , MT)

13: endif

14: endfor

15: return MT

### 2.2 GateMeClass training module

The training module can be executed using the R function *GateMeClass_training*. It inputs an annotated cytometry dataset in the form of an expression matrix (Algorithm 1, line 1) and outputs a marker table with a pseudo-gating strategy for each cell type (Fig. 1, top left panel and Algorithm 1, line 15). This module executes the routine that generates a marker signature for each cell (Algorithm 1, lines 2–4) and uses this information to extract a minimal set of markers critical to classify each cell type. After creating an empty marker table (Algorithm 1, line 5), for each pair of cell types $\left(c_{i},\;c_{j}\right)$ (Algorithm 1, lines 6–14), GateMeClass trains a classification tree using the R package “caret” (Kuhn 2008) and calculates the marker importance measuring the goodness of split in which the variable was a primary or surrogate (Breiman *et al.* 1984) (Algorithm 1, lines 7–8). For each $\left(c_{i},\;c_{j}\right)$, the training routine follows a greedy strategy selecting always the most discriminant marker $M_{k}\;$(Fig. 1 bottom left panel, Algorithm 1, line 9). Then, the entries of $c_{i}$ and $c_{j}$ in the marker table are updated with the values ($M_{k}$+), ($M_{k}$m), or ($M_{k}-$) according to the percentage of the cells $c_{i}$ and $c_{j}$ that have high, medium, or low expression of $M_{k}$ (Algorithm 1, lines 10–13). It is worth underlining that the classification tree will consider the increasing priority ($M_{k}-$) < ($M_{k}$m)< ($M_{k}$+) to determine the most discriminating marker. Our greedy strategy keeps the set of parameters for each cell type in the marker table to a minimum to avoid the use of nondiscriminating markers. To do this, we update the table entries of a pair $\left(c_{i},\;c_{j}\right)$ only if the top discriminant marker between $c_{i}$ and $c_{j}$ is not already present in the current marker table. In the training routine, the default behavior of GateMeClass is to model the marker expression as “−“ or “+.” To make GateMeClass able to manage medium expression, there must be almost three labels containing the name of a marker plus each of the suffixes “−,” “mid,” and “hi.” For example, in the presence of monocytes CD11b-, CD11bmid, and CD11bhi, the label of the corresponding cells should be “monocytes_CD11b-,” “monocytes_CD11bmid,” and “monocytes_CD11bhi.”

Algorithm 2. Annotation module of GateMeClass.

1: GateMeClass_annotation(dataset, MT):

2: dataset’ ← SRS(dataset, p)

3: D ← Parse_Marker_Table(MT)

4: for each cell c_i_ in dataset’:

5: S_i_ ← Cell_Signature(c_i_ )

6: endfor

7: A ← Cell_Classification(dataset’, S, D)

8: if reject_option = TRUE:

9: Create A’ and U’ using MNN

10: A’’ ← kNN_Refinement(dataset, A’∨U’, U-U’)

11: else:

12: A’’ ← kNN_Refinement(dataset, A, U)

13: endif

14: return A’’

### 2.3 GateMeClass annotation module

The annotation module of GateMeClass can be executed using the R function *GateMeClass_annotation*. It inputs a dataset in the form of a matrix and a marker table (Algorithm 2, line 1) and returns a vector of cell annotations (Fig. 1 top right panel and Algorithm 2, line 14). The first (optional) step of annotation is the random sampling of the cells (Algorithm 2, line 2). The user can choose to classify only a subset of the cells and annotate the rest through the refining procedure based on k-NN. Our rationale is that only a small subset is needed to annotate a cluster of cells and the rest can be annotated by similarity. Next, the marker table is loaded and parsed to extend the pseudo-gating strategies in the actual possible definitions of a cell type (Algorithm 2 line 3). In fact, in addition to the option of defining a marker with “*” meaning the marker can be “+,” “m,” or “−,” GateMeClass also enables conditional marker expressions. Conditions on marker expression are defined using the operators “|” and “^.” For example, to define that a monocyte can express CD11b, CD11c, or both, the user could specify the entry CD11b+|CD11c+|. Furthermore, the user can be interested in monocytes expressing CD11b or CD11c but not both using the entry CD11b+^CD11c+^. After parsing the marker table, the marker signature is defined for each cell (Algorithm 1, lines 4–6). The actual annotation step is performed by comparing the cell signatures with the entries of the marker table. Both “*” and logical operators can cause ambiguity in the annotation phase. Therefore, prior to classification, an ordered dictionary of possible signatures is created for each cell type, which, in the case of a tie, prioritizes the cell annotation with a greater number of markers with high or medium expression in the signature. After the annotation step (Algorithm 2, line 7), if some cells remain unclassified, a two-step refinement process is performed using (i) mutual nearest neighbors (MNNs) (optional) and (ii) k-NN classification (Fig. 1, bottom right panel and Algorithm 2, lines 8–13). In our context, two cells are MNNs if each cell is in the k-nearest neighbor, in the Euclidean space, of the other. The MNN procedure is used to help the identification of new cell subsets not defined in the marker table and depends on the optional parameter “reject_option.” Given the set of annotated and unclassified (or unsampled) cells *A* and *U*, respectively, if the parameter “reject_option” is set to false, the k-NN classification is executed directly using training set *A* and control set *U*, otherwise the MNN procedure is executed. The aim of using MNNs is to create a high-confidence training set to make the k-NN algorithm able to distinguish real unknown cell subsets from cells marked as unclassified due to classification errors. In particular, the training set will be composed by a set *A*′ of cells confidently annotated with a cell type and a set *U*′ of confident unknown cells. To this end, MNN is used, first, to detect potential classification errors to be removed from the annotated cells. We assume that annotated cells with a higher proportion of MNNs in the set of unclassified cells are ambiguous and must be reannotated. The set *A*′ will be the set of annotated cells excluding the ambiguous ones. Second, the set *U*′ of high confident unknown cells will be composed of those unclassified cells with no MNNs in the set of annotated cells. The general idea is that the real unknown cell subsets tend to cluster together and are more similar compared to other annotated cell types. Next, k-NN classification is executed using as training set the *A*′ ∨ *U*′ and control set *U—U*′.

### 2.4 Evaluation metrics

We used four evaluation metrics to measure the performance of GateMeClass: (i) the overall accuracy, (ii) the precision, (iii) the recall, and (iv) the F1-score. The overall accuracy is calculated as the percentage of correct classifications (true positive TP, true negative TN) over the total number of classifications (true positive TP, true negative TN, false positive FP, false negative FN):

(2) $$\mathrm{Accuracy}\left(\%\right)=\frac{TP+TN}{TP+TN+FP+FN}\times 100$$

For each reference label, the precision is calculated as the number of true positive over the number of true positive plus false positive:

(3) $$\mathrm{Precision=}\frac{TP}{TP+FP}$$

For each reference label, the recall is calculated as the number of true positive over the number of true positive plus false negative:

(4) $$\mathrm{Recall}=\frac{TP}{TP+FN}$$

For each reference label, the F1-score was calculated using the following formula:

(5) $$F1=\frac{2\times \mathrm{precision}\times \mathrm{recall}}{\mathrm{precision}+\mathrm{recall}}$$

All the metrics were calculated using the R package “caret.”

## 3 Results

To test the ability of GateMeClass to annotate cytometry data, we obtained three well-established manually gated benchmark mass cytometry datasets and one flow cytometry dataset. The mass cytometry datasets were obtained from the R package “HDCytoData” (Weber and Soneson 2019): AML (Levine *et al.* 2015), BMMC (Levine *et al.* 2015), and PANORAMA (Samusik *et al.* 2016) from 2 healthy bone marrow donors, 1 healthy bone marrow donor, and 10 mouse bone marrows, respectively. The AML dataset is composed of 104 184 cell events and 32 markers (CD45RA, CD133, CD19, CD22, CD11b, CD4, CD8, CD34, Flt3, CD20, CXCR4, CD235ab, CD45, CD123, CD321, CD14, CD33, CD47, CD11c, CD7, CD15, CD16, CD44, CD38, CD13, CD3, CD61, CD117, CD49d, HLA-DR, CD64, CD41), the BMMC is composed of 81 747 cell events and 13 markers (CD45, CD45RA, CD19, CD11b, CD4, CD8, CD34, CD20, CD33, CD123, CD38, CD90, CD3), PANORAMA is composed of 514 386 cell events and 39 surface marker proteins (Ter119, CD45.2, Ly6G, IgD, CD11c, F480, CD3, NKp46, CD23, CD34, CD115, CD19, 120g8, CD8, Ly6C, CD4, CD11b, CD27, CD16_32, SiglecF, Foxp3, B220, CD5, FceR1a, TCRgd, CCR7, Sca1, CD49b, cKit, CD150, CD25, TCRb, CD43, CD64, CD138, CD103, IgM, CD44, MHCII). The flow cytometry dataset is composed of three samples from a published study (Bost *et al.* 2021). This dataset is characterized, respectively, by 29 193, 23 472, and 21 453 cell events with the following protein markers: FS-A, SS-A, CD57, CD19, CD45RA, CD8, CD56, CD4, CD27, CD45, CD3, CD16. For each cytometry dataset, we measured the performance of GateMeClass in terms of accuracy, precision, recall, and F1-score.

### 3.1 Evaluation and setting of the GateMeClass parameters for the classification of cytometry data

**Figure 2.**
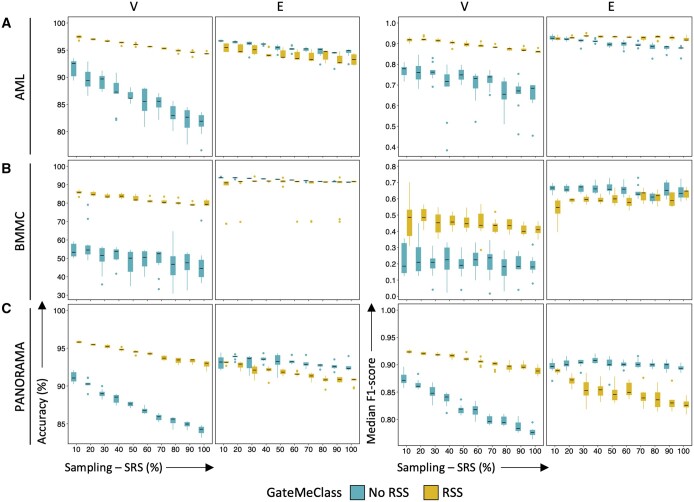
Performance evaluation. GateMeClass was tested on (A) AML, (B) BMMC, and (C) PANORAMA datasets. Each box plot represents the performance distribution of 10 random executions for each configuration of sampling (10%–100%) and variance parameters (V and E). The sampling percentage is a GateMeClass parameter (optional) used to perform the classification of a subset of a dataset before completing the annotation of the rest using the refinement process. Accuracy (first two columns) and median F1-score (last two columns) are shown. Precision and recall are reported in Supplementary Tables S1–S3

To evaluate the different parameter configurations of GateMeClass, for each mass cytometry dataset, we measured the performance of GateMeClass varying its parameters: sampling (SRS) percentages (10%–100%), GMM variance parameterizations (i.e. E or V), and with or without RSS (Fig. 2). Each dataset was split into training (50%) and control (50%) and, for each configuration, 10 random executions were performed by varying the training and control sets. The results show that sampling the dataset before annotating all the cells is not associated with worse performance; conversely, using only the 10% of the cells results in the best accuracy in many cases (Fig. 2). Therefore, this sampling percentage was set as the default configuration of GateMeClass and was considered for subsequent comparisons. The performance of GateMeClass is affected by the choice of the GMM variance parameter and the use of RSS. In fact, the best choice of the GMM parameter depends on the properties of the dataset and, in particular, on the variance (equal or variable/unequal) of the expected Gaussian components composing the distribution of each marker. This is, in turn, influenced by cell composition of the sample, fluorochromes, optical properties of the detection system and cell cycle. GateMeClass V is positively affected by RSS, which leads to a significant improvement in terms of accuracy and median F1-score compared to the no-RSS counterpart. Conversely, GateMeClass E is negatively affected by RSS (Fig. 2, Supplementary Fig. S1A). In AML, GateMeClass E and GateMeClass V with RSS achieved comparable performance but the latter achieved an overall accuracy of 97.9% with a median F1-score of 0.93 while GateMeClass E achieved 97% accuracy with a median F1-score of 0.95 (Fig. 2A, Supplementary Table S1). In GateMeClass V, the RSS method led to significant performance improvement because it provides a clearer picture of the shape of the distribution of each marker, emphasizing bimodality in most parameters (Supplementary Fig. S1B). In BMMC, GateMeClass E clearly outperformed GateMeClass V, achieving an accuracy of 94.3% and a median F1-score of 0.68 compared to 87.4% and 0.67 for GateMeClass V (Fig. 2B, Supplementary Table S2). In PANORAMA, GateMeClass V achieved the best results obtaining an accuracy of 96% and F1-score of 0.93 compared to 94.4% and 0.91 of GateMeClass E (Fig. 2C, Supplementary Table S3). In terms of single-cell labels, in all three datasets, progenitor cells (e.g. MEP, CLP) that were present in very low percentages (<1%) generally obtained a lower F1-score than mature cells, while hematopoietic stem cells (HSCs) and multipotent progenitors (MPPs) were not detected in BMMC (Supplementary Fig. S1C). Overall, the results show that in GateMeClass, the choice of the variance parameterization is important and depends on the biological and technical properties of the dataset. Furthermore, the RSS improves only the performance of GateMeClass V. The latter performed better than GateMeClass E in AML and PANORAMA but showed a drop in performance in BMMC. Conversely, GateMeClass E achieved over 90% of accuracy in all the datasets.

### 3.2 Comparison of GateMeClass and marker table-based and reference set-based classification tools

GateMeClass was compared with two classes of tools for cytometry data: classification tools based on the definition of a marker table, ACDC and MP (Lee *et al.* 2017, Ji *et al.* 2018), and tools based on a reference set of annotated cells, DGCyTOF and LDA (Abdelaal *et al.* 2019, Cheng *et al.* 2022). All the tests were executed on a MacBook Pro equipped with an Intel^®^ Core i7 2.6 GHz processor and 16 GB RAM and with R version 4.2.1. For the first comparison, the ACDC marker table, with few changes in PANORAMA (Supplementary Table S4), was used for all the tools. For the second comparison, the AML and BMMC datasets were split into training (50%) and control (50%). For all the tools, the default configuration was used. Similar to ACDC, and for a sake of comparability with all the methods, erythroblasts, megakaryocytes, platelets, myelocytes, and monocytes with CD11b^mid^ were merged as an unknown population in BMMC. In PANORAMA, HSCs and pro-B cells were merged as an unknown population.

In AML, GateMeClass and ACDC behaved similarly and outperformed MP in terms of overall accuracy while MP achieved the highest median F1-score (Table 1). In the reference-based setting, DGCyTOF was the best-performing tool in terms both of accuracy (99.3%) and median F1-score (0.98), while GateMeClass and LDA obtained comparable results.

**Table 1.** The performance of GateMeClass in terms of accuracy (A) and median F1-score (F1) on benchmark mass cytometry datasets.

Classification using a marker table							Reference-based classification					
	GateMeClass		ACDC		MP		GateMeClass		DGCyTOF		LDA	
Dataset	A(%)	F1	A(%)	F1	A(%)	F1	A(%)	F1	A(%)	F1	A(%)	F1
AML	97.9	0.93	98.4	0.93	93.1	0.94	97.9	0.93	99.3	0.98	98.2	0.94
BMMC	93.2	0.66	94	0.70	93.5	0.61	94.3	0.68	98.9	0.95	95.7	0.85
PANORAMA	93.8	0.84	95.4	0.94	71.0	0.57	96.0	0.93	99.8	0.99	97.0	0.93

GateMeClass was tested both using the ACDC marker table and dividing each dataset into training (50%) and control (50%). For each tool, the best run, in terms of accuracy, among 10 random executions was reported. Each tool was executed using the default setting.

In BMMC, GateMeClass outperformed MP in terms of median F1-score, while ACDC obtained the highest levels of accuracy and median F1-score (Table 1). In the reference-based setting and similar to the AML dataset, DGCyTOF obtained the most accurate results (98.9% of accuracy and 0.95 median F1-score) followed by LDA. In BMMC, GateMeClass obtained greater accuracy and higher median F1-score in the reference-based setting (94.3% of accuracy and 0.68 median F1-score) compared to the classification based on the marker table (93.2% of accuracy and 0.66 median F1-score). Of note, in BMMC GateMeClass was executed with “reject_option= T” to detect the unknown population that was not defined in the ACDC marker table.

**Table 2.** Execution time of GateMeClass and the other tools on benchmark datasets.

	Classification using a marker table			Reference-based classification		
Dataset	GateMeClass	ACDC	MP	GateMeClass	DGCyTOF	LDA
AML	36.28 s	707.61 s	6057.69 s	61.98 s	16.69 s	6.52 s
BMMC	20.82 s	140.05 s	1514.59 s	33.27 s	12.24 s	1.641 s
PANORAMA	763.0 s	15 219 s	56 077 s	711.80 s	94.35 s	39.48 s

The times reported refer to the best configurations reported in Table 1.

To test the ability of GateMeClass to manage the medium expression of a marker, we tried to execute GateMeClass without merging monocytes CD11b^mid^ into the set of unknown cells, obtaining an F1-score of 0.63 for that population and validating the ability of GateMeClass to detect cells with medium marker expression.

In PANORAMA, GateMeClass and ACDC performed better than MP both in terms of accuracy and median F1-score. GateMeClass and ACDC obtained comparable overall accuracy, 93.8% and 95.4%, respectively, while ACDC achieved the highest median F1-score (0.94). Similarly to BMMC, GateMeClass was executed with “reject_option= T.” In the reference-based setting and similarly to the other benchmark datasets, DGCyTOF reached both the best accuracy (99.8%) and median F1-score (0.99), while GateMeClass and LDA obtained comparable performance. In terms of execution time, GateMeClass was the fastest tool among the marker table-based methods, classifying the AML dataset in 36.28 s, BMMC in 20.82 s, and PANORAMA in 763 s (Table 2); while among the reference-based tools, LDA was the fastest, classifying the three benchmark datasets in few seconds. Compared to the marker table-based tools, GateMeClass achieved comparable or better performance. The advantage of GateMeClass is that the marker table can be obtained automatically from data and without using external sources. In addition, GateMeClass is faster and, unlike ACDC and MP, is able to manage the medium expression of a marker. In the automatic setting, compared to the reference-based classification tools, GateMeClass performed well, reaching 97.9%, 94.3%, and 96% of accuracy on AML, BMMC, and PANORAMA. Despite the good accuracy and execution time of GateMeClass (∼62 s in AML, ∼33 s in BMMC, and ∼712 s in PANORAMA), in this setting DGCyTOF and LDA performed better and were faster (Table 2). However, the advantage of GateMeClass compared to the reference-based tools is the ability to annotate cytometry data without a reference set of annotated cells, which is not always available.

In the flow cytometry dataset, we performed manual gating to obtain annotations. The following cell populations were identified: neutrophils (67%), CD4^+^ (22.2%) and CD8^+^ T cells (2.1%), monocytes (7%), NK cells (0.9%), and B cells (0.8%) (Supplementary Fig. S2). To demonstrate the ability of the tool to annotate cytometry data using an independent training dataset, we extracted the marker table from a published study on a cohort of COVID-19 patients (Bronte *et al.* 2020) using the training module of GateMeClass. To obtain comparable cell labels between the two datasets, we collapsed the more specific cell subtypes of the training set into broader categories, e.g. CD4^+^ and CD8^+^ T effector memory (TEM) cells were merged into CD4^+^ and CD8^+^ T cell labels, and cell types absent in the actual dataset were removed from the marker table obtained by GateMeClass. Prior to classification, the .fcs files of the three control samples were compensated, transformed using the Logicle transformation (Parks *et al.* 2006) and concatenated in one dataset.

**Figure 3.**
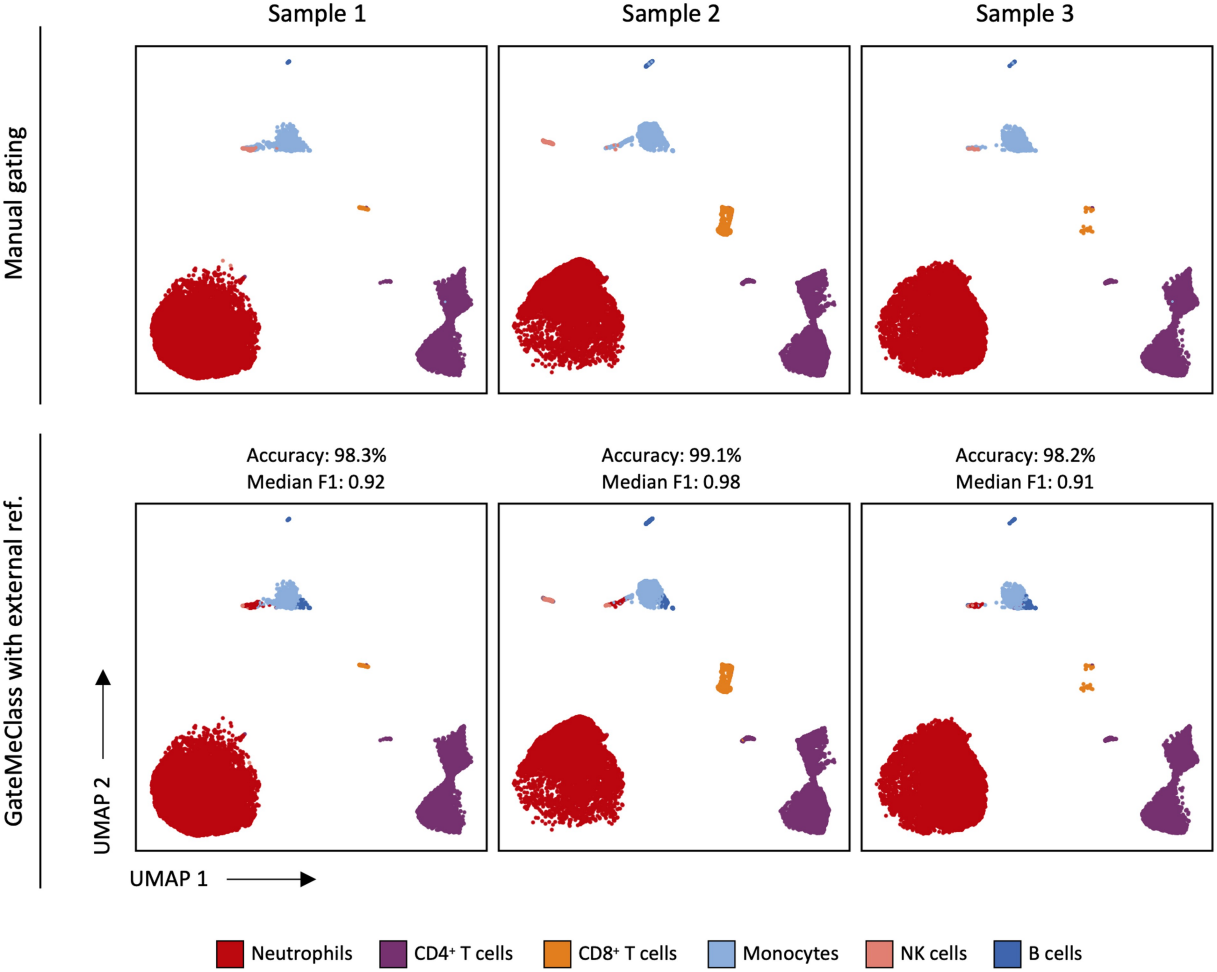
Performance of GateMeClass on manually gated flow cytometry data. UMAP plots showing the classification results of GateMeClass on three flow cytometry samples using an external reference (Bronte *et al.* 2020). The figure shows the overall accuracy and the median F1-score for each sample

The results of the classification show that GateMeClass was able to annotate the flow cytometry datasets as well as manual gating (Supplementary Fig. S2), with minor errors mainly on monocytes and NK cells, obtaining 98.8% of accuracy and a median F1-score of 0.96 (Table 3, Fig. 3). In this analysis, we used GateMeClass with an equal variance parameter. For comparison purposes, we also tested DGCyTOF and LDA on this dataset. The results were comparable among the three tools in terms of overall accuracy (∼99%), while DGCyTOF and LDA obtained a slightly higher median F1-score and faster execution times (Table 3).

**Table 3.** Execution time of GateMeClass on flow cytometry dataset.

	GateMeClass			DGCyTOF			LDA		
Dataset	A (%)	F1	Time (s)	A (%)	F1	Time (s)	A (%)	F1	Time (s)
FC dataset	98.8	0.96	31.93	99.3	0.98	17.52	99	0.98	1.94

Overall, the results show the ability of GateMeClass to annotate cytometry data with accuracy and execution times comparable to the other state-of-the-art methods, while offering greater flexibility of use. In fact, GateMeClass can be executed with a manually defined marker table or with a reference annotated dataset that can be used as a training set. Furthermore, compared to the marker table-based tools, GateMeClass is faster, and it offers the option of identifying cell types characterized by medium expression.

## 4 Conclusion

GateMeClass is a new tool for the automatic annotation of cytometry data. Our tool is able to annotate the cell populations using a marker table that can be defined manually or extracted from an external annotated dataset in a reference-based fashion. In addition, unlike the other marker table-based classification tools, GateMeClass manages medium expression by bypassing the binary definition of a marker as positive or negative. One limitation of GateMeClass is that the training module is not able to estimate how many mixture components should be used for a given marker. In fact, by default, GateMeClass uses the binary definition of a marker and medium expression must be explicitly guided by the user. Furthermore, the best variance parameterization must be carefully chosen by the user looking at the data distribution and with testing. In fact, the choice of E or V depends on the variance of the expected Gaussian components underlie the distribution of each marker. A future direction will be to enable GateMeClass to automatically select, for each marker, the GMM variance parameter and the number of mixture components.

## Supplementary Material

btae322_Supplementary_Data
