# Supplementary material for: GateMeClass: Gate Mining and Classification of cytometry data
Source: Bioinformatics. 2024 May 22;40(5):btae322. doi: 10.1093/bioinformatics/btae322 (PMC11136448; doi:10.1093/bioinformatics/btae322)
Supplement: btae322_Supplementary_Data [file btae322_supplementary_data.zip › Caligola_et_al_Supp.docx]

**GateMeClass: Gate Mining and Classification of cytometry data**

**Supplemental data**

**Supplementary Fig. 1**

(A) Overall accuracy (%) and median F1-score of GateMeClass E and V with or without RSS on the biological replicates of the datasets tested in Fig. 2 (sampling of 10%). Paired Wilcoxon rank-sum test was used to assess statistical significance (***p< 0.0001, **p<0.001). (B) Distribution of the marker intensities calculated by GateMeClass with or without RSS on AML. (C) Single labels F1-score obtained by GateMeClass on (left panel) AML, (central panel) BMMC and (right panel) PANORAMA datasets. The red lines denote the median F1-score.

**Supplementary Fig. 2**

Manual gating strategy used to annotate the cells of the flow cytometry dataset showed in Fig. 3.
